# Association between epilepsy and challenging behaviour in adults with intellectual disabilities: systematic review and meta-analysis

**DOI:** 10.1192/bjo.2020.96

**Published:** 2020-09-25

**Authors:** Shoumitro Deb, Basma Akrout Brizard, Bharati Limbu

**Affiliations:** Faculty of Medicine, Department of Brain Sciences, Imperial College London, UK; Institut de Psychologie, Laboratoire de Psychopathologie et Processus de Santé, Paris, France; Faculty of Medicine, Department of Brain Sciences, Imperial College London, UK

**Keywords:** Intellectual disabilities, adults, challenging behaviour, systematic review, meta-analysis

## Abstract

**Background:**

Previous systematic reviews showed no significant association between epilepsy and challenging behaviours in adults with intellectual disabilities.

**Aims:**

To identify whether there is an association between epilepsy and challenging behaviour in adults with intellectual disabilities by carrying out a systematic review of published data. PROSPERO registration number: CRD42020178092.

**Method:**

We searched five databases and hand-searched six journals. Two authors independently screened titles, abstracts and full articles using a standardised eligibility checklist. Several meta-analyses were carried out.

**Results:**

The narrative analysis of data from 34 included articles (14 168 adults with intellectual disabilities, 4781 of whom also had epilepsy) showed no significant association between epilepsy and challenging behaviour. Meta-analysis was possible on data from 16 controlled studies. This showed no significant intergroup difference but after sensitivity analysis meta-analysis of 10 studies showed a significantly higher rate of overall challenging behaviour in the epilepsy group (effect size: 0.16) compared with the non-epilepsy group. Aggression and self-injurious behaviour both showed a statistically significant higher rate in the epilepsy group, with very small effect sizes (0.16 and 0.28 respectively). No significant intergroup difference was observed in the rate of stereotypy.

**Conclusions:**

The findings are contradictory and must be interpreted with caution because of the difficulty in pooling data from varied studies, which is likely to introduce confounding. Where significant differences were found, effect sizes are small and may not be clinically significant, and there are major methodological flaws in the included studies, which should be addressed in future large-scale properly controlled studies.

Epilepsy is common in adults with intellectual disabilities, with an average estimated point prevalence of 25%, compared with <1% in the general population who do not have intellectual disabilities.^1^ The rate increases with the severity of disability:^2^ around 7–15% in mild to moderate intellectual disability, 67% in severe intellectual disability and 82% in profound intellectual disability. The rate increases if the intellectual disability is associated with other neurological disorders, such as cerebral palsy.^1^

## Special issues relating to epilepsy in adults with intellectual disabilities

Certain genetic syndromes that lead to intellectual disabilities, such as Angelman, Sturge–Weber, fragile-X/ataxia, Rett, Lesch–Nyhan, Rubinstein–Taybi, Lowe and Down syndromes and tuberous sclerosis, are commonly associated with epilepsy.^3^ A high proportion of people with autism spectrum disorder (22%) also have epilepsy.^1^ Similarly, certain epilepsy syndromes, such as West syndrome (in infants), Lennox–Gastaut syndrome, Landau–Kleffner syndrome and Dravet syndrome, are more commonly associated with intellectual disabilities.^4^ Compared with the general population of adults who do not have intellectual disabilities, epilepsy among adults with intellectual disabilities is not only more prevalent, but it also often manifests as multiple seizure types, starts at an early age, is of longer duration and is resistant to anti-epileptic treatment (in over 30% in the general population, compared with over 70% in intellectual disabilities).^5^ Diagnosing epilepsy and seizure type can be difficult in this population, and both false-positive (stereotypy, cardiac syncope, non-epileptic attack disorder may all mimic epileptic seizure) and false-negative (difficulty diagnosing absence, focal seizures) diagnoses are possible.^6^ Also, these people are more prone to die from sudden unexpected death in epilepsy (SUDEP).^7^^,^^8^

## Relationship between epilepsy and challenging behaviour in adults with intellectual disabilities

The relationship between epilepsy and challenging behaviour in adults with intellectual disabilities is complex.^9^ Challenging behaviour has been defined as ‘socially unacceptable behaviour that causes distress, harm or disadvantage to the persons themselves or to other people, and usually requires some intervention’.^10^ Challenging behaviour is prevalent among adults with intellectual disabilities, affecting up to around 62%.^11^^–^^14^ More severe forms of challenging behaviour are manifested by a lower proportion (18.7–30%).^13^^,^^15^ The types of challenging behaviour include aggression, destruction of property, disruptive behaviour, self-injurious behaviour, stereotypy, and sexually inappropriate and harmful behaviours.^14^^,^^16^ Aggression is reported in 10–20% of adults with intellectual disabilities.^16^^,^^17^ The aetiology of challenging behaviour is multifactorial, including medical, psychiatric, psychological, social and environmental factors. Therefore, a multiprofessional approach is required to formulate a person-centred treatment plan for this difficult-to-manage problem.^16^ Demographic factors such as age, gender, severity of intellectual disability, associated comorbidities (e.g. other neurodevelopmental disorders such as autism spectrum disorder, attention-deficit hyperactivity disorder), medical conditions and psychosocial factors can all affect challenging behaviour in adults with intellectual disabilities.^14^^,^^18^ Epilepsy is one such factor that may influence a person's behaviour.

## Previous systematic reviews on the subject

Three systematic reviews looked at the association between challenging behaviour and epilepsy in people with intellectual disabilities: none of these found any association.^19^^–^^21^ We decided to carry out an updated systematic review, as important publications either have appeared since the last reviews or were not included in those reviews. Another reason for carrying out this review is to conduct meta-analyses that were not done in any of the previous reviews.

## Method

The aim of the current systematic review and meta-analysis is to identify the rates and types of challenging behaviour in adults with intellectual disabilities who have epilepsy (‘the epilepsy group’) and compare them with rates and types in adults with intellectual disabilities who do not have epilepsy (‘the non-epilepsy group’) to determine whether epilepsy is a risk factor for developing challenging behaviour in this population.

We aimed to include studies that compared the overall rate of challenging behaviour as well as different types of challenging behaviour in adults with intellectual disabilities with and without epilepsy within the same cohort. We did not aim to include any study that involved only adults with intellectual disabilities who did not have epilepsy. We included studies that involved participants without epilepsy only where they were part of the control group and participants with epilepsy were also involved in the same study.

We also aimed to include studies that involved adults with intellectual disabilities and epilepsy but no one without epilepsy. These studies are included to assess the rate of different types of challenging behaviour according to different seizure variables.

### Search strategy

We decided on our final search strategy after an initial scoping literature search. We followed PROSPERO guidelines^22^ and the PRISMA-P checklist to develop our protocol and search strategy.^23^ Five electronic databases – Embase, PubMed/MEDLINE, PsycInfo, DARE and ASSIA (ProQuest) – were searched for relevant journal articles. Each database was searched between 1 January 1985 and 31 May 2020. In addition, we also cross-referenced pertinent reviews and articles. We hand-searched for relevant articles in the past 20 years’ issues, from January 1990 to May 2020, in the following intellectual disabilities journals: (a) *Journal of Intellectual Disability Research*, (b) *Journal of Applied Research in Intellectual Disabilities* (*JARID*) and (c) *Research in Developmental Disabilities*; and the following epilepsy journals: (a) *Seizure*, (b) *Epilepsy & Behavior* and (c) *Epilepsia*.

Only quantitative studies in English were searched. We excluded non-human studies, studies involving children and conference abstracts.

### Search terms

The list of search terms used can be found in the supplementary material available at https://doi.org/10.1192/bjo.2020.96. Search terms were adapted from previous systematic reviews that were carried out to develop a national and an international guide for the use of psychotropic medications in the management of challenging behaviour in adults with intellectual disabilities.^10^^,^^24^

### Criteria for selecting studies

We devised a list of eligibility criteria based on PROSPERO^22^ and Cochrane review guidelines^25^ and adapted from similar systematic reviews on psychotropic medications^26^^–^^28^ (supplementary Appendix 1).

### Types of study

There was no restriction on the type of study design included in this review. We included both randomised and non-randomised studies; controlled and non-controlled observational or cross-sectional studies; and controlled studies with both matched and non-matched control groups.

### Types of participant

All participants had intellectual disabilities, were aged 16 years or over and displayed various types of challenging behaviour. We have only included data on challenging behaviour in this review. Data on psychiatric illness without challenging behaviour are not presented in the current paper as they will be included in a separate systematic review article.

No ethical approval was required for this study as no individual patient-related data were collected or analysed.

### Sample size

Studies with fewer than 10 participants were excluded. This arbitrary cut-off was used in accordance with our previous systematic reviews.^10^

### Secondary outcome

The rates of different types of challenging behaviour (verbal aggression and aggression towards people or property or self, stereotypy, overactivity, temper tantrum, etc.) were compared between the epilepsy and the non-epilepsy groups. Data on subgroup comparisons according to different types of seizure, seizure frequency and different pharmacological regimes (e.g. polypharmacy versus monopharmacy of anti-epileptic medications; treatment with carbamazepine versus valproate) were collected to identify the role of different epilepsy-related factors in the development of challenging behaviour.

### Selection process

**Fig. 1:**
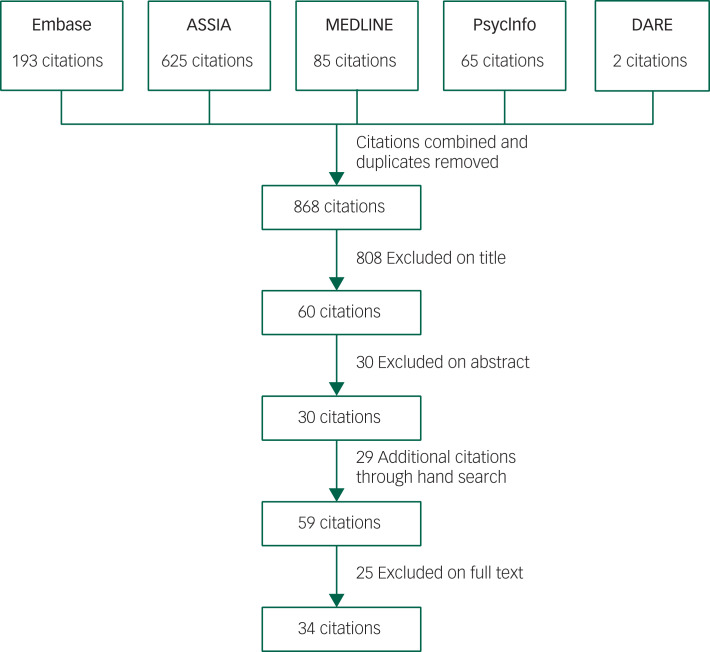
The flowchart of the paper selection process.

After the definitive search was completed, titles were searched for key terms. Zotero reference management software^29^ was used to manage and record references from each database. Duplicates, non-human studies and studies involving children were identified by Zotero and removed manually (by B.A.B.). The remaining abstracts were screened independently using the pre-piloted eligibility criteria (by B.A.B. and B.L.). The two authors were masked to each other's scores. Bibliographies of potential studies were screened to identify articles that required acquisition of the full text. Discrepancies identified were reviewed and discussed between B.A.B. and B.L. to resolve any differences. The full texts were then reviewed and independently assessed for eligibility by B.A.B. and B.L. using the same eligibility checklist that was used for screening the abstracts. The selection process is shown in Fig. 1. It was not necessary for the third review author (S.D.) to arbitrate.

### Data extraction

Data from studies meeting eligibility criteria were extracted independently by B.A.B. and B.L. using a standardised data extraction template adapted from Cochrane review guidelines (supplementary Appendix 2).^30^ S.D. checked this information for accuracy. Results are reported using a narrative synthesis of information from included studies.

### Meta-analysis

The meta-analysis was carried out only on controlled studies. An odds ratio was calculated for the studies that presented the proportion of participants in each group displaying challenging behaviour. A standardised mean difference was calculated for those studies that presented means and standard deviations for scores based on a behaviour rating scale in the two groups.^31^ To pool data, we log-transformed all data to standardised mean differences.^31^ RevMan 5.3^32^ software for Windows 10 was used for random-effects meta-analysis. Heterogeneity was tested using the χ2-test and *I*^2^-statistic. Where there was substantial heterogeneity (*I*^2^ > 60%), a further sensitivity analysis was carried out. A final meta-analysis was carried out after removing data from the studies that produced a high heterogeneity and strong bias, to bring heterogeneity to an acceptable level (*I*^2^ < 30).

### Risk-of-bias assessment and confidence in cumulative estimates

The risk of bias for the 19 controlled studies identified was assessed independently by B.A.B. and B.L. using the Cochrane risk-of-bias tool^33^ and the quality of all 32 eligible studies was assessed during the data collection process using the SIGN 50 checklist.^34^

Publication bias was assessed using a funnel plot, and the studies included were assessed for consistency and precision. The quality of the overall systematic review was assessed using AMSTAR 2 criteria (see Appendix 3; supplementary material).^35^

## Results

### Included studies

Of the 868 articles screened, 34 papers (from 32 studies) met the eligibility criteria and were included in the systematic review (Fig. 1). A list of excluded articles with reason for exclusion is presented in supplementary Appendix 4. Two studies each published two papers on the same sample but on different outcome measures. Of the 34 papers, 19 included participants with and without epilepsy, as their authors compared the rates of challenging behaviour in these two groups to assess an association between challenging behaviour and epilepsy (Table 1). Of these, nine studies^36^^–^^44^ had equal numbers of participants in both groups and the groups were matched on various demographic variables. The rest (*n* = 10)^12^^,^^45^^–^^53^ were prevalence studies of challenging behaviour in adults with intellectual disabilities that included a number of participants with epilepsy (around 22% of the cohort). Different variables, such as age, gender and presence/absence of epilepsy, were used to assess whether they are risk factors for developing challenging behaviour. The rates of challenging behaviour were compared on the basis of the presence or absence of epilepsy, but the epilepsy groups were not matched with the non-epilepsy groups. The rest of the studies included participants with epilepsy only, and in these the rates of challenging behaviour were compared between various types of epilepsy (e.g. frequent versus infrequent; generalised versus focal seizures). Table 2 compares the rates of different types of challenging behaviour (e.g. aggression, self-injurious behaviour) between the epilepsy and the non-epilepsy groups. Table 3 presents data on participants with epilepsy only and compares rates of challenging behaviour according to various epilepsy variables. Most studies were carried out in the UK (*n* = 24), some in the USA (*n* = 4) and one each in Ireland, Sweden, The Netherlands and Spain. In total, data on 14 168 adults with intellectual disabilities are presented (4781 with epilepsy and 9387 without epilepsy).

**Table 1:** Rates of challenging behaviour in adults with intellectual disabilities with and without epilepsy

Study and country	Sample, control group	Participant age, years	Measures used	Statistical analysis	Results
Studies with matched control group					
Deb et al (1987),^36^ UK	32 with epilepsy; 32 without epilepsy	16–70	DAS severity ≥3	Student's *t*-test	No statistically significant intergroup difference in maladaptive behaviour scores (epilepsy group, mean: 21.7, s.d. = 4.5; non-epilepsy group, mean: 20.3, s.d. = 4.8)
Espie et al (1989),^37^ UK	15 with epilepsy; 15 without epilepsy	20–46	ABS-II (maladaptive behaviour), PBS	Wilcoxon matched-pairs signed-ranks test	No statistically significant intergroup difference according to the ABS-II total score (epilepsy group, mean: 75.53, s.d. = 9.02; non-epilepsy group, mean: 72.4, s.d. = 7.91). PBS subscores and factor scores did not show any intergroup significant difference either
Gillies et al (1989),^38^ UK	65 with epilepsy; 65 without epilepsy	Mean: 30.1	PBS	Student's *t*-test	Statistically significant higher total PBS score in the epilepsy group compared with the control group (epilepsy group, mean: 41.99, s.d. = 24.31; non-epilepsy group, mean: 30.2, s.d. = 22.4, *P* = 0.005)
Collacott (1993),^39^ UK	Down syndrome: 35 with epilepsy; 68 without epilepsy	Under 20 to over 60	ABS-II (maladaptive behaviour)	ANOVA	No significant intergroup difference in total ABS-II score
Deb (1997),^40^ UK	150 with epilepsy; 150 without epilepsy	20–77	PAA (maladaptive behaviour items)	Wilcoxon matched-pairs signed-ranks test	No statistically significant difference in severe maladaptive behaviour between epilepsy group (58%) and non-epilepsy group (52%)
Matson et al (1999),^41^ USA	353 with epilepsy; 353 without epilepsy	Mean: 37.8	DASH-II, ABC	ANOVA	Epilepsy group had a statistically significant lower DASH-II total score and ABC total score compared with the non-epilepsy group. DASH-II total score (epilepsy group, mean: 11, s.d. = 10.58; non-epilepsy group, mean: 15.2, s.d. = 11.22, *P* < 0.001). ABC total score (epilepsy group, mean: 12.4, s.d. = 16.43; non-epilepsy group, mean: 21.37, s.d. = 21.54, *P* < 0.001)
Chung & Cassidy (2001),^42^ UK	Part 2: 14 with epilepsy; 14 without epilepsy	Mean: 35	ABC subdomains	Student's *t*-test	Epilepsy group had a statistically significant higher ABC-Irritability score (mean: 17.86) compared with the non-epilepsy group (mean: 8.2) (*t* = 2.99, *P* < 0.01). No significant intergroup difference was found in any other ABC subscores
Matthews et al (2008),^43^ UK	55 with epilepsy; 55 without epilepsy	17–86	ABC total score >45	χ^2^	No significant intergroup difference (epilepsy group: 23.6% *v.* non-epilepsy group: 20%).
Smith & Matson (2010),^44^ USA	25 with epilepsy; 25 without epilepsy	17–86	ASD-CA	MANOVA and ANOVA	No statistically significant intergroup difference in conduct disorder score (epilepsy group, mean: 1.4, s.d. = 1.58; non-epilepsy group, mean: 1.16, s.d. = 2.19) (*F* = 1.22; p = 0.31)
Studies with unmatched control group					
Prasher (1995),^45^ UK	201 with Down syndrome, of whom 32 had epilepsy	Mean: 44.2	ABS-II (maladaptive behaviour) (compared between 12 participants with epilepsy and 47 without)	Not stated (possibly Student's *t*-test)	No significant intergroup difference in total ABS-II score (epilepsy group, mean: 18.27, s.d. = 9.10; non-epilepsy group, mean: 15.84, s.d. = 10.40 (*t* = 0.16))
Deb et al (2001),^12^ UK	101, including 25 with epilepsy	16–64	DAS	χ^2^	No significant intergroup difference in the rate of overall maladaptive behaviour (76% of epilepsy group; 55% of non-epilepsy group)
Espie et al (2003),^46^ UK	178 with epilepsy compared with 1022 normative non-epilepsy standardisation group	Mean: 35.5	ABC subdomains	Compared scores with normative data using Cronbach alpha	All ABC subdomain scores in the epilepsy group were consistently 4 points lower (equivalent to 0.5 s.d.) compared with the normative data. All scores were consistently below 50th percentile. ABC-Irritability sample mean: 6.54, s.d. = 7.6 *v*. normative data mean 10.54, s.d. = 9.8
Turkistani (2004),^47^ UK	108 with epilepsy; 132 without epilepsy	Mean: 40.3	Parents’ and carers’ report of disturbed behaviour	χ^2^	No significant difference in the rate of disturbed behaviour between epilepsy (27.7%) and non-epilepsy (37.9%) groups
McGrother et al (2006),^48^ UK	2393, including 620 with epilepsy	Over 20	DAS	Univariate, multivariate Logistic regression	People with epilepsy had significantly higher total DAS score than those without epilepsy (54.2 *v.* 42%, OR = 1.6, *P* < 0.0001)
Pawar & Akuffo (2008),^49^ UK	177, including 53 with epilepsy	Over 17	Case notes	Descriptive statistics	Lower rates of challenging behaviour in the epilepsy group (59%) compared with the non-epilepsy group (70%)
Fitzgerald et al (2011),^50^ USA	321 with severe and profound intellectual disabilities, including 115 with epilepsy’	20–88	DASH-II	MANCOVA followed by ANCOVA and Bonferroni correction (*P* < 0.004)	No intergroup statistically significant difference in DASH-II items apart from mood subscale
O'Dwyer et al (2018),^51^ Ireland	676, including 216 with epilepsy	Over 40	Authors' own problem behaviour questionnaire	Regression analysis	56% in the epilepsy group and 51.3% in the non-epilepsy group displayed challenging behaviour. This difference was statistically non-significant
Folch et al (2018),^52^ Spain	833, including 242 with epilepsy	18–84	ABC-C SIB items	χ^2^	30.4% of those who displayed SIB had epilepsy and 28.8% of those without SIB had epilepsy. This difference was not significant
Blickwedel et al (2019),^53^ UK	238 with challenging behaviour, including 70 with epilepsy	Epilepsy group: mean 40.2, s.d. = 14.2; non-epilepsy group: mean 38, s.d. = 14.7	ABC-C total score	Student's *t*-test	Epilepsy group had a lower total ABC-C score, but the intergroup difference was not significant (epilepsy group; mean: 61.8, s.d. = 28.6; non-epilepsy group; mean: 67.0, s.d. = 28.8; *P* = 0.204)

ABC-C, Aberrant Behaviour Checklist-Community version; ABS-II, Adaptive Behaviour Scale Part II; ANOVA, analysis of variance; ANCOVA, analysis of covariance; ASD-CA, Autism Spectrum Disorders-Comorbidity-Adult version battery; DAS, Disability Assessment Schedule; DASH-II, Diagnostic Assessment for the Severely Handicapped-Part 2; MANOVA, multivariate analysis of variance; MANCOVA, multivariate analysis of covariance; PAA, Profile of Abilities and Adjustment schedule; PBS, Psychosocial Behaviour Scale; SIB, self-injurious behaviour.

**Table 2:** Types of challenging behaviour in adults with intellectual disabilities with and without epilepsy

Study and country	Study design	Sample, control group	Age, years	Measures used	Statistical analysis	Results
Gillies et al (1989),^38^ UK	Matched controlled study	65 with epilepsy; 65 without epilepsy	Mean: 30.1	PBS	Student *t*-test	Significantly higher rates of physical aggression in the epilepsy group compared with the control group *(t* = 2.02, d.f. = 40, *P* < 0.05)
Espie et al (1989),^37^ UK	Matched controlled study	15 with epilepsy; 15 without epilepsy	20–46	ABS-II (maladaptive behaviour)	Wilcoxon matched-pairs signed-ranks test	ABS-II SIB score was significantly lower (mean: 76.40, s.d. = 10.12, *v.* mean: 86.20, s.d. = 11.35, *F* = −2.49, *P* = 0.006) but aggressive and destructiveness scores were similar (mean: 74.26, s.d. = 19.80, *v.* mean: 74.33, s.d. = 20.76, *F* = −0.16, *P* = 0.438) in the epilepsy group compared with the non-epilepsy group
Creaby et al (1993),^54^ UK	Unmatched controlled study	230 with severe intellectual disability, including 131 epilepsy	Mean: epilepsy 38.8; non-epilepsy 40.3	Information gathered from case notes using a checklist	χ^2^	37.4% of epilepsy and 36.3% of non-epilepsy groups showed paroxysmal aggression. This difference was not statistically significant
Collacott et al (1998),^58^ UK	Cross-sectional study	2101, of whom 372 showed SIB. Number with epilepsy is not known	Mean: 33.56	DAS	Mann–Whitney	No difference in the prevalence of epilepsy between those with and without SIB (χ^2^ = 2.36, d.f. = 1, *P* = 0.13)
Andrews et al (1999),^67^ UK	Cross-sectional study	115 with epilepsy	18–93	ABC subdomain scores	Mann–Whitney	Scores for all 5 ABC subdomains (irritability, lethargy, stereotype, hyperactivity, inappropriate speech) are lower than the normative data when matched for intellectual disability level
Deb et al (2001),^12^ UK	Observational study	101, including 25 with epilepsy	16–64	DAS	Regression analysis	Severe challenging behaviour was significantly associated with epilepsy (40 *v.* 18.4%; χ^2^ = 4.83, d.f. = 1, *P* = 0.02). Physical aggression (epilepsy group: 36% *v.* non-epilepsy group: 18.4%) and SIB (epilepsy group: 32% *v.* non-epilepsy group: 21%) showed no significant intergroup difference
McGrother et al (2006),^48^ UK	Population-based study	2393, including 620 with epilepsy	Over 20	DAS	Logistic regression with Bonferroni correction	No significant difference in individual item scores such as physical aggression (15.3 *v.* 11.7%), destructive behaviour (13.5 *v.* 9.1%), excessive activity (6.7 *v.* 4.3%), SIB (11.7 *v.* 7.3%) and antisocial behaviour (4.2 *v.* 6.1%). Epilepsy group showed significantly higher rate compared with non-epilepsy group of (a) disturbing others at night (12.2 *v.* 5.9%, OR = 2.20, *P* < 0.0001), (b) seeking attention (16 *v.* 10%, OR = 1.62, *P* < 0.001) and (c) being uncooperative (20 *v.* 12.6%, OR = 1.74, *P* < 0.0001)
Tyrer et al (2006),^59^ UK	Cross-sectional design	3062, including 812 with epilepsy	19–92	DAS	Logistic regression	19% of people with epilepsy showed aggressive behaviour compared with 13% without epilepsy (OR = 1.55, *P* < 0.001). Physical aggression to others was not significantly associated with epilepsy (OR = 0.95, *P* = 0.67)
Pawar & Akuffo (2008),^49^ UK	Observational survey	177, including 53 with epilepsy	Over 17	Case notes	Descriptive statistics	Verbal aggression: 11% in epilepsy group *v*. 35% in non-epilepsy group. Physical assault: 35% in the epilepsy group *v*. 34% in non-epilepsy group. Inappropriate sexual behaviour: 2% in epilepsy group *v*. 6% in non-epilepsy group
Cooper et al (2009),^73^ UK	Prospective cohort study	1023, including 349 with epilepsy	Over 16	DC-LD	Univariate, multivariate regression analysis	SIB: 7.2% in epilepsy group *v*. 3.5% in non-epilepsy group (*P* = 0.009). At multivariate analysis this difference was not significant
Cooper et al (2009),^74^ UK	Prospective cohort study	1023, including 349 with epilepsy	Over 16	DC-LD	Univariate, regression analysis	No statistically significant intergroup difference in aggressive behaviour between epilepsy (12%) and non-epilepsy (8.4%) groups
Smith & Matson (2010),^77^ USA	Observational study	100, including 25 with epilepsy	29–76	ASD-BPA	MANOVA and ANOVA	No significant intergroup (epilepsy *v.* non-epilepsy) difference in aggressive behaviour (mean: 0.68 *v.* 0.56), stereotypy (mean: 0.68 *v.* 0.52), SIB (mean: 0.2 *v.* 0.08) and disruptive behaviour (mean: 0.2 *v.* 0.44)
Lundqvist (2013),^13^ Sweden	Observational study	915, including 124 with epilepsy	18–87	BPI with additional questions	Univariate and multivariate regression analysis	SIB: 45.2% in epilepsy group *v.* 28.7% in non-epilepsy group; stereotypical behaviour: 50 *v.* 39.9%; aggressive/destructive behaviour: 37.1 *v.* 34%. None of these differences were statistically significant after multivariate regression analysis
Blickwedel et al (2019),^53^ UK	Unmatched controlled study	238 with challenging behaviour, including 70 with epilepsy	Epilepsy group, mean: 40.2, s.d. = 14.2; non-epilepsy group, mean: 38	ABC-C subdomains	χ^2^	None of the ABC-C subdomain scores related to challenging behaviour showed any significant intergroup difference. Irritability (epilepsy group, mean: 21.1, s.d. = 11.6 *v.* non-epilepsy group, mean: 20.3, s.d. = 10.1, *P* = 0.588); stereotypy (mean: 5.3, s.d. = 4.3, *v.* mean: 6.4, s.d. = 5.3, *P* = 0.143); hyperactivity (mean: 19.3, s.d. = 10, *v.* 20.1, s.d. = 9.9, *P* = 0.58)

ABC-C, Aberrant Behaviour Checklist-Community version; ABS-II, Adaptive Behaviour Scale Part II; ANOVA, analysis of variance; ASD-BPA, Autism Spectrum Disorders-Behaviour Problem-Adult version; BPI, Behavior Problem Inventory; DAS, Disability Assessment Schedule; DC-LD, Diagnostic Classification-Learning Disability; MANOVA, multivariate analysis of variance; PBS, Psychosocial Behaviour Scale; SIB, self-injurious behaviour.

**Table 3:** Challenging behaviours in adults with intellectual disabilities according to different epilepsy variables

Reference	Study design	Sample, control group	Age, years	Measures used	Statistical analysis	Results
Gillies et al (1989),^38^ UK	Unmatched controlled study	21 with high frequency of seizures; 44 with low frequency of seizures	Mean: 30.1	PBS	Student's *t*-test	Statistically significant higher total PBS score in the high seizure-frequency group (*n* = 21) (mean: 54.05, s.d. = 24.57, *t* = 2.92) compared with the low seizure-frequency group (*n* = 44) (mean: 36.23, s.d. = 22.22, *t* = 2.92, *P* = 0.005)
Espie et al (1989),^37^ UK	Matched controlled study	15 with epilepsy; 15 without epilepsy	20–46	ABS-II (maladaptive behaviour), PBS	ANCOVA	ABS-II antisocial behaviour domain score significantly higher in the high seizure-frequency group (mean: 82.83, s.d. = 18.71 *v.* mean: 60.55, s.d. = 23.73, *F* = 2.38, P = 0.045) but not PBS physical aggression domain score (mean: 9.66, s.d. = 3.98 *v.* mean: 5.33, s.d. = 5.36, *F* = 2.85, *P* = 0.058) compared with the lower seizure-frequency group. AED polypharmacy (compared with AED monopharmacy) was significantly associated with ABS II violent and destructive behaviour score (mean: 87.16, s.d. = 7.73 *v.* mean: 65.66, s.d. = 21, *F* = 4.52, *P* = 0.033), but not with PBS physical aggression (mean: 9.66, s.d. = 5.82 *v.* mean: 5.33, s.d. = 4.18, *F*: 1.7, *P* = 0.01)
Espie et al (1990),^69^ UK	Observational study	65 with epilepsy	Mean: 30.33	PBS total score	Rank sum test (non-parametric comparison of median values)	Global behaviour problems (PBS total score) were greater in the AED polypharmacy group (mean: 52.3, s.d. = 23.9) than in the AED monopharmacy group (mean: 33.5; s.d. = 19.7) (*P* < 0.001)
Deb & Hunter (1991),^61^ UK	Matched controlled study	150 with epilepsy; 150 without epilepsy	20–77	PAA (maladaptive behaviour items)	Wilcoxon	Among in-patients: the epilepsy group showed significantly (a) less aggression in the single seizure group and less irritability in the EEG group who had only slow background wave compared with epileptiform activities, (b) more irritability in the EEG group who had generalised epileptiform activity, (c) less aggression in the AED monopharmacy group and the carbamazepine monopharmacy group. Among community patients: SIB was associated with multiple and frequent seizures
Creaby et al (1993),^54^ UK	Observational study	131 with severe intellectual disability and epilepsy	Mean: 40	Case notes	χ^2^	41.7% with generalised seizures and 17.4% with partial seizures showed aggression (χ^2^ = 4.74, d.f. = 1, *P* = 0.029); 32% of those who received polypharmacy and 41.3% of those who received AED monopharmacy showed aggression (this difference was non-significant); 35% with severe (very frequent) seizures, 41% with moderately frequent seizures, and 40% with mild (infrequent) seizures showed aggression (these differences were not significant)
Deb (1995),^62^ UK	Observational study	100 with epilepsy	20–77	PAA (maladaptive behaviour items)	χ^2^	58% of those with generalised epileptiform changes in EEG as opposed to 50% of those with focal changes in EEG displayed maladaptive behaviour. This difference was not statistically significant
Collacott et al (1998),^58^ UK	Cross-sectional study	372 with SIB; 1729 without SIB	Mean: 33.56	DAS	χ^2^	No significant difference in seizure frequency between SIB and the non-SIB group (χ^2^ = 2.36, d.f. = 1, *P* = 0.13)
Deb & Joyce (1999),^55^ UK	Retrospective study	143 with epilepsy	20–83	Case notes; purpose-designed proforma	χ^2^	Statistically significant higher rate of challenging behaviour among those with GTCS (63%) than those without GTCS (31%) (χ^2^ = 5.9, d.f. = 1, *P* = 0.01). No intergroup difference in any other seizure type, seizure frequency or type of AED use
Deb & Joyce (1999),^56^ UK	Retrospective study	143 with epilepsy	20–83	Case notes; purpose-designed proforma	χ^2^	46% with challenging behaviour compared with 42% without such behaviour received AED polypharmacy. This difference was not statistically significant Among AED monopharmacy, 59% of those receiving carbamazepine, 55% sodium valproate, 53% phenytoin and 78% receiving lamotrigine showed challenging behaviour. None of these differences were significant
Andrews et al (1999),^67^ UK	Observational study	116 with epilepsy	18–93	ABC subdomain scores	χ^2^	Participants with generalised seizures (median: 8.5) had non-significantly higher ABC subscores than those with partial seizures (median: 1), apart from hyperactivity and inappropriate speech, which were significantly higher (hyperactivity subscore; χ^2^ = 11.4, d.f. = 2, *P* = 0.003; inappropriate speech subscore; χ^2^ = 6, d.f. = 2; *P* = 0.048). No significant associations were found between the category of MRI abnormalities (focal *v.* diffuse cerebral *v.* no lesion) and the ABC factors No significant association between ABC scores and number and type of AED prescribed
Espie et al (2003),^46^ UK	Cross-sectional study	178 with epilepsy compared with 1022 normative non-epilepsy standardisation group	Mean: 35.5	ABC subdomains	Compared scores with normative data using Cronbach's alpha	ABC scores were most strongly related to non-epilepsy-specific concerns such as intellectual, sensory or motor function rather than epilepsy-specific concerns such as seizure severity and frequency
Turkistani (2004),^47^ UK	Unmatched controlled study	108 with epilepsy; 132 without epilepsy	Mean: 40.3	Parents’ and carers’ report of disturbed behaviour	χ^2^	Seizure frequency did not have a significant effect on the rate of challenging behaviour. No significant difference in the rate of challenging behaviour between those who received AED monopharmacy *v*. those who received AED polypharmacy
Ring et al (2007),^57^ UK	Observational study	110 with active epilepsy (at least one seizure in the past 3 months); 65 with non-active epilepsy (no seizures in the past 3 months)	16–72	Case notes and carer interviews	χ^2^	No statistically significant difference in the rate of challenging behaviour between active epilepsy (19%) and non-active epilepsy group (18%). SIB was displayed by 7% of the non-active and 9% of the active epilepsy groups. This difference was not statistically significant
van Ool et al (2018),^72^ Netherlands	Cross-sectional design	189 with epilepsy	18–85	BPI (Dutch version)	Regression analysis	SIB significantly associated with a lower number of AED (OR = 0.54, *P* = 0.018). Stereotyped behaviour significantly associated with a higher number of seizure types (OR = 1.56, *P* = 0.001), a higher seizure frequency (OR = 1.02, *P* = 0.015), a lower number of AEDs (OR = 0.70, *P* = 0.048). Aggressive/destructive behaviour was significantly associated with a higher number of seizure types (OR = 1.42, *P* = 0.006)
Blickwedel et al (2019),^53^ UK	Unmatched controlled study	238 with challenging behaviour, including 70 with epilepsy	Epilepsy group, mean: 40.2; non-epilepsy group, mean: 38	ABC-C	Multiple regression analysis	No significant difference in ABC-C scores between those with focal and primary generalised seizures or between those receiving AED mono- or polypharmacy

ABC-C, Aberrant Behaviour Checklist-Community version; ABS-II, Adaptive Behaviour Scale Part II; AED, anti-epileptic drugs; ANCOVA, analysis of covariance; BPI, Behaviour Problem Inventory; DAS, Disability Assessment Schedule; EEG, electroencephalogram; MRI, magnetic resonance imaging; PAA, Profile of Abilities and Adjustment schedule; PBS, Psychosocial Behaviour Scale; SIB, self-injurious behaviour; GTCS, generalised tonic–clonic seizures.

### Diagnosis

Challenging behaviour is defined using a variety of methods in different studies. Five studies^49^^,^^54^^–^^57^ collected data retrospectively from participants’ case notes and did not use any validated measures. One study^47^ collected information on challenging behaviour from parents’ and carers’ reports and one study^51^ used its own questionnaire, for which no validation data are provided. Among the studies that used validated questionnaires, five^12^^,^^36^^,^^48^^,^^58^^,^^59^ used challenging behaviour items from the Disability Assessment Schedule (DAS)^60^ and another three^40^^,^^61^^,^^62^ used DAS challenging behaviour items from the Profile of Abilities and Adjustment schedule (PAA).^61^^,^^63^ Three studies^37^^,^^39^^,^^45^ used the Adaptive Behaviour Scale Part II (ABS-II),^64^ which scores maladaptive behaviours. Two studies^41^^,^^50^ used the Diagnostic Assessment for the Severely Handicapped Part 2 (DASH-II).^65^ Two studies^43^^,^^53^ used the Aberrant Behaviour Checklist-Community version (ABC-C) total score,^66^ and another four^42^^,^^46^^,^^52^^,^^67^ used ABC-C subdomain scores.^68^ Three studies^37^^,^^38^^,^^69^ used the Psychosocial Behaviour Scale (PBS).^70^ The Behaviour Problem Inventory (BPI)^71^ was used in two studies^12^^,^^72^ to rate challenging behaviour. One study each used the Diagnostic Criteria-Learning Disability (DC-LD),^73^^–^^75^ Autism Spectrum Disorders-Comorbidity-Adult version (ASD-CA)^44^^,^^76^ and Autism Spectrum Disorders-Behaviour Problems for Adults (ASD-BPA)^77^^,^^78^ respectively.

### Statistical methods used

Where a standardised scale was used to measure challenging behaviours, some studies compared means and standard deviations for the two groups, whereas others used an arbitrary cut-off score to compare participants in the two groups.^79^ Various statistical methods were used, including χ^2^, regression analysis, univariate and multivariate regression analyses, the Wilcoxon matched-pairs signed-ranks test and Student's *t*-test.

### Outcome (narrative synthesis)

#### The overall rate of challenging behaviour

Of the total 19 controlled studies, 13^12^^,^^36^^,^^37^^,^^39^^,^^40^^,^^43^^–^^45^^,^^47^^,^^50^^–^^53^ did not show any significant intergroup difference in the overall rate of challenging behaviour, three^38^^,^^42^^,^^48^ showed a significantly higher rate of challenging behaviour in the epilepsy group and three^41^^,^^46^^,^^49^ showed a higher overall rate of challenging behaviour in the non-epilepsy group (Table 1). Of these three, one^41^ was at a significant level and the level of significance for other two^46^^,^^49^ is not known.

#### Rates of different types of challenging behaviour

Table 2 shows the rates of different types of challenging behaviour in the epilepsy and the non-epilepsy groups. For aggression, nine studies^12^^,^^13^^,^^37^^,^^48^^,^^49^^,^^53^^,^^54^^,^^74^^,^^77^ showed no significant intergroup difference. According to two studies^38^^,^^59^ the epilepsy group showed a statistically significant higher rate of aggression compared with the non-epilepsy group. In one study^67^ aggression was less common in the epilepsy group. For self-injurious behaviour, six studies^12^^,^^13^^,^^48^^,^^58^^,^^73^^,^^77^ showed no significant intergroup difference and one study^37^ showed significantly less self-injurious behaviour in the epilepsy group compared with the non-epilepsy group. For aggression to property or destructiveness, three studies^13^^,^^37^^,^^48^ showed no significant intergroup difference and no study showed a higher rate in the epilepsy group compared with the non-epilepsy group. One study^48^ showed a significantly higher rate of behaviours such as disturbing others at night, seeking attention and being uncooperative in the epilepsy group compared with the non-epilepsy group. Rates of behaviours such as stereotypy, inappropriate sexual behaviour, irritability, hyperactivity, verbal aggression, antisocial behaviours and lethargy. were reported in a very small number of studies showing equivocal findings.

#### Association between challenging behaviour and epilepsy-related variables

Table 3 presents data on the rate of challenging behaviour according to different epilepsy variables. For example, in three studies^54^^,^^61^^,^^72^ a significantly higher rate is reported among those with generalised seizures as opposed to focal seizures but in one study^67^ the intergroup difference was not significant. Similarly, three studies^38^^,^^61^^,^^72^ showed a higher rate of challenging behaviour among those who presented with frequent seizures as opposed to those who had less frequent seizures. However, in five studies^37^^,^^47^^,^^54^^,^^55^^,^^58^ no such significant intergroup difference was found. In one study^61^ those who showed generalised epileptiform changes on electroencephalograms (EEGs) showed a significantly higher rate of challenging behaviour compared with those whose EEGs showed focal epileptiform changes.

#### Association between challenging behaviour and anti-epileptic medication-related variables

Three studies^37^^,^^61^^,^^69^ showed a significantly higher rate of challenging behaviour among those who received multiple anti-epileptic medications (polypharmacy group) compared with those who received single anti-epileptic medication (monopharmacy group). However, no such significant intergroup difference was found in five studies.^37^^,^^47^^,^^53^^,^^54^^,^^56^ Interestingly, two studies^54^^,^^72^ reported a higher rate of challenging behaviour in the monopharmacy group than in the polypharmacy group, and in one of these studies this intergroup difference was significant.^72^ Only one study reported the rate of challenging behaviour in various monopharmacy groups (phenytoin, sodium valproate, carbamazepine and lamotrigine monopharmacy) but it made no intergroup comparison.^56^

### Meta-analysis

**Fig. 2:**
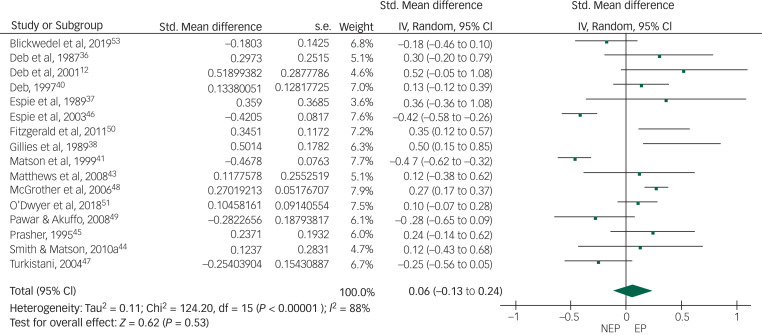
Forest plot of total challenging behaviour score data from 16 studies. NEP, no epilepsy; EP, epilepsy.

**Fig. 3:**
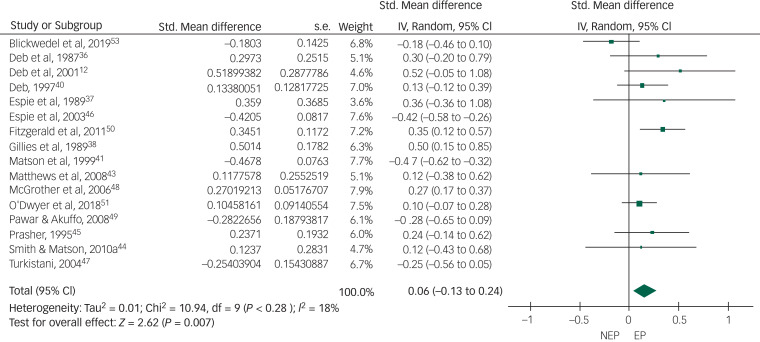
Forest plot of total challenging behaviour score data from 10 studies after sensitivity analysis. NEP, no epilepsy; EP, epilepsy.

**Fig. 4:**
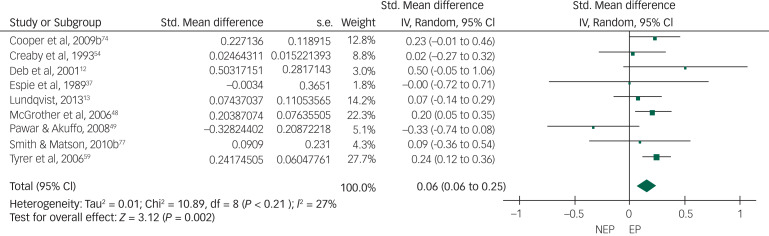
Forest plot of aggression score data. NEP, no epilepsy; EP, epilepsy.

**Fig. 5:**
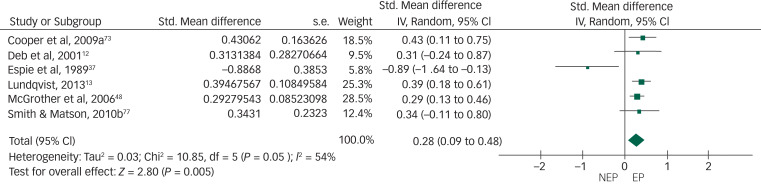
Forest plot of self-injurious behaviour score data. NEP, no epilepsy; EP, epilepsy.

**Fig. 6:**
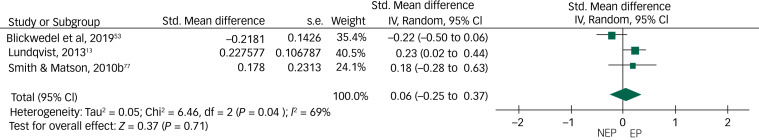
Forest plot of stereotypy score data. NEP, no epilepsy; EP, epilepsy.

Of the 19 controlled studies, data were available for meta-analysis from 16. Pooled data from the 16 studies using a random-effects meta-analysis of standardised mean differences showed no significant intergroup difference but the heterogeneity among studies was very high (*I*^2^ = 88%) (Fig. 2). After sensitivity analysis we removed data from studies that produced the highest level of heterogeneity and had a high risk of bias according to the Cochrane risk-of-bias tool. The final meta-analysis, using a random-effects model, of pooled data from the remaining 10 studies showed a statistically significant higher rate of overall challenging behaviour in the epilepsy group compared with the non-epilepsy group, with a very small effect size of 0.16. The heterogeneity among studies came down to an acceptable level (*I*^2^ = 18%) (Fig. 3). To study specific types of challenging behaviour, we conducted meta-analyses of pooled data on aggression scores from nine studies (Fig. 4), on self-injurious behaviour from six (Fig. 5) and on stereotypy from three (Fig. 6). The aggression meta-analysis showed a significantly higher rate in the epilepsy group compared with the non-epilepsy group, with a very small effect size of 0.16. The heterogeneity level was low (*I*^2^ = 27%). The self-injurious behaviour meta-analysis also showed a statistically significant higher rate in the epilepsy group compared with the non-epilepsy group, with a very small effect size of 0.28, but the heterogeneity score was high, albeit below 60% (*I*^2^ = 54%). The stereotypy meta-analysis did not show any significant intergroup difference but showed a high heterogeneity value of over 60% (*I*^2^ = 69%).

### Quality control

**Fig. 7:**
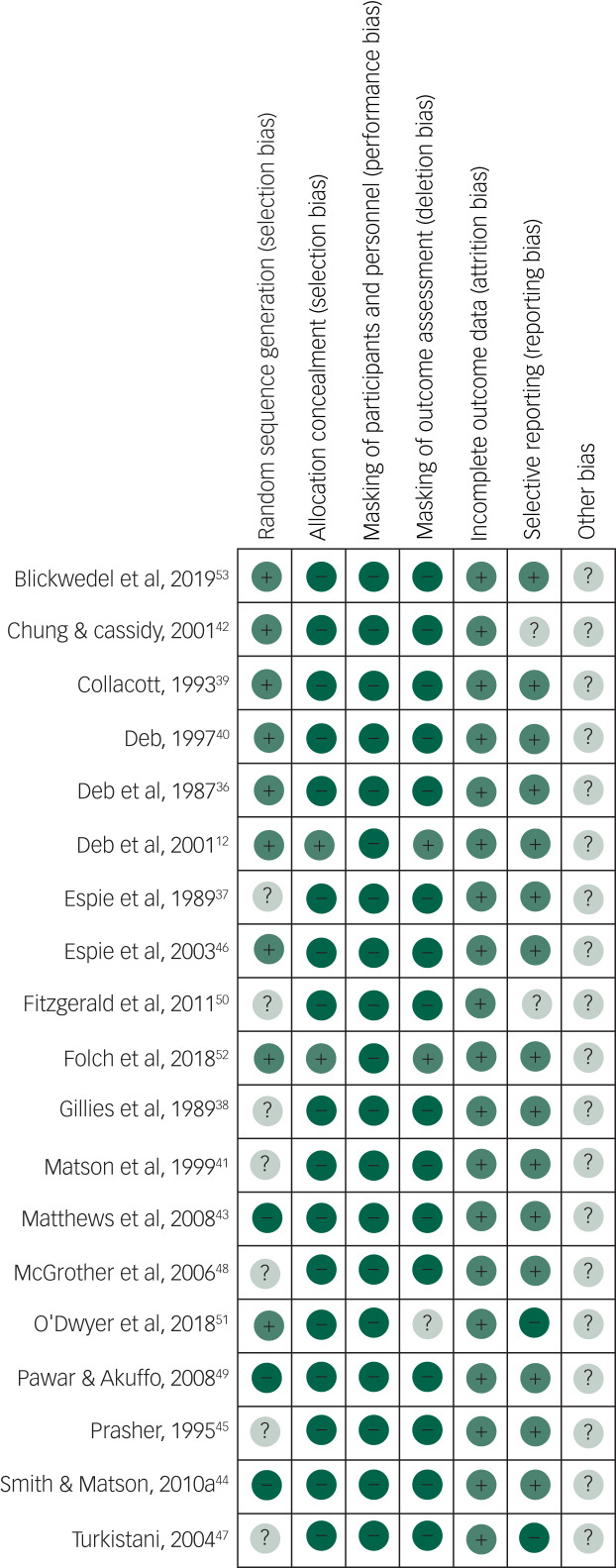
Cochrane risk-of-bias summary for the 19 controlled studies. +, bias present; −, bias absent; ?, bias possible.

The AMSTAR2 checklist showed a high score, indicating that this systematic review and meta-analysis is of a high standard (supplementary Appendix 3). The SIGN 50 checklist identified only 5 of the 32 studies to be of high quality and the Cochrane risk-of-bias assessment of the 19 controlled studies showed a high risk of bias in most domains for most of the studies (Fig. 7). A summary graph is presented as supplementary Appendix 5. Publication bias could not be identified using a funnel plot for stereotypy, as data from only three studies could be amalgamated into the meta-analysis. Funnel plot data showed no publication bias for aggression, which was further supported by Egger's test of publication bias (*P* = 0.213).^80^ Although the funnel plot showed some publication bias for overall rate of challenging behaviour, this was not significant under Egger's test of publication bias (*P* = 0.734). There was no publication bias present for self-injurious behaviour (*P* = 0.307). Eleven articles reported receiving funding from external sources, one did not receive any funding and the rest (*n* = 22) did not declare the funding source.

## Discussion

We included 34 articles in our systematic review that met the eligibility criteria. These included 9 studies that compared the overall rate of challenging behaviour in an epilepsy group with a matched control group and another 10 that compared data with an unmatched control group. Compared with previous systematic reviews this review included data for a much higher number of participants (Table 4).

**Table 4:** Comparison of systematic reviews

Systematic review	Number of participants	Study characteristics	Findings
de Winter et al (2011)^20^	Only 8 out of 45 studies included participants with epilepsy. Total participants: not reported (5263 children and adults with epilepsy and matched controls, calculated from the study characteristics Table)	Search strategy: English, Dutch or German language studies published between January 1990 and July 2008 with a minimum sample size of five participants. Included studies: 45 papers involving children and adults with intellectual disabilities, only 8 of which included participants with epilepsy. All physical and medical conditions were included. All levels of intellectual disability were included. Outcome: Main aim was to find prevalence of different medical conditions, including epilepsy. Within that context, challenging behaviour was compared between those with and without epilepsy in 8 studies only. Data analysis: Only narrative synthesis, no meta-analysis	No significant association was found between epilepsy and challenging behaviour. People with more severe and frequent seizures may be at a higher risk of developing challenging behaviour
van Ool et al (2016)^21^	461 children and adolescents, 7742 adults and elderly, and 392 participants of mixed age groups. Total number of participants with epilepsy was not reported	Search strategy: English language studies published between January 1995 and January 2015. Included studies: 15 papers involving children and adults with intellectual disabilities (9: presence and absence of epilepsy in people with intellectual disabilities; 4: differences in epilepsy-related factors in people with intellectual disabilities and epilepsy; 4: presence or absence of intellectual disabilities in people with epilepsy). All types of epilepsy and all levels of intellectual disability were included. Outcome: Neuropsychiatric comorbidity in general, no separate data on challenging behaviour. Data analysis: Only narrative synthesis, no meta-analysis	Presence of epilepsy only is not a clear determinant of neuropsychiatric comorbidity in people with intellectual disabilities. More severe forms of epilepsy had an association, but most included studies showed a high risk of bias
Blickwedel et al (2019)^19^	5653 participants, including 2032 with epilepsy	Search strategy: English language studies published between January 1985 and August 2016 with a minimum sample size of five participants. Included studies: 25 articles involving adults only with any level of intellectual disability. Outcome: Overall challenging behaviour, different types of challenging behaviour, challenging behaviour according to different epilepsy variables. Data analysis: Only narrative synthesis, no meta-analysis	No significant association between epilepsy and overall challenging behaviour. No significant association between overall challenging behaviour and aggression, self-injurious behaviour and stereotypy. Significantly higher rate of irritability was associated with epilepsy. More frequent seizures were associated with self-injurious behaviour and antisocial behaviour. Limited evidence that generalised epilepsy may be associated with challenging behaviour
Deb et al (2020) (current review)	14 168 adults with intellectual disabilities, including 4781 adults with epilepsy	Search strategy: English language articles published between January 1985 and May 2020. Included studies: 34 articles based on 32 studies involving adults with intellectual disabilities only. 19 controlled studies (9 with and 10 without a matched control group). Outcome: Overall challenging behaviour, different types of challenging behaviour and challenging behaviour according to different epilepsy variables (seizure frequency, generalised *v*. focal seizures, generalised *v*. focal EEG changes, polypharmacy *v*. monopharmacy with anti-epileptics). Data analysis: Narrative synthesis and meta-analysis	The narrative analysis of data from 34 included articles showed no significant association between epilepsy and challenging behaviour. No definite association was found in the rate of challenging behaviour and different epilepsy variables, such as frequency of seizures, generalised *v*. focal epilepsy, polypharmacy of anti-epileptic drugs (narrative analysis only). Meta-analysis was possible on pooled data from only 16 controlled studies. This showed no significant intergroup difference but, after sensitivity analysis, pooled data from 10 studies showed a significantly higher rate of overall challenging behaviour in the epilepsy group (effect size: 0.16) compared with the non-epilepsy group. Aggression and self-injurious behaviour both showed a statistically significant higher rate in the epilepsy group, with a very small effect size (0.16 and 0.28 respectively). No significant intergroup difference was observed in the rate of stereotypy

In our review, of the total 19 controlled studies, 13^12^^,^^36^^,^^37^^,^^39^^,^^40^^,^^43^^–^^45^^,^^47^^,^^50^^–^^53^ did not show any significant intergroup difference in the overall rate of challenging behaviour, 3^38^^,^^42^^,^^48^ showed a significantly higher rate of challenging behaviour in the epilepsy group and 3^41^^,^^46^^,^^49^ showed a higher rate in the non-epilepsy group. Of these three, one^41^ was at a significant level and the level of significance for the other two^46^^,^^49^ is not known.

It was possible to pool data from only 16 of the controlled studies for a meta-analysis. Meta-analysis of pooled data from these 16 studies did not show a significant intergroup difference in the overall rate of challenging behaviour but showed a high heterogeneity. However, after sensitivity analysis meta-analysis of pooled data from 10 studies showed a significantly higher rate of overall challenging behaviour in the epilepsy group compared with the non-epilepsy group. Meta-analysis of pooled data showed a statistically significant higher rate of aggression and self-injurious behaviour in the epilepsy group compared with the non-epilepsy group. However, no such intergroup difference emerged from the meta-analysis of pooled stereotype data.

A funnel plot and Egger's test of publication bias showed no publication bias among the included studies. According to both SIGN 50 and Cochrane risk-of-bias assessments most studies appeared to be of moderate to poor quality.

### Interpretation of meta-analyses findings

Our finding (based on meta-analysis of pooled data from 10 studies) of a significant intergroup difference differs from that of other systematic reviews,^19^^–^^21^ which found no such difference. Our finding has to be interpreted with caution. First, when data from all available studies were pooled, no statistically significant intergroup difference emerged, although the heterogeneity among studies was very high. Second, even when the meta-analysis after the sensitivity analysis showed that the epilepsy group had a significantly higher rate of challenging behaviour, the effect size remained very small, so this may not be clinically significant. Third, different studies defined challenging behaviour in different ways. Some did not use any validated tool. Fourth, even when a validated scale was used, the total score was used to define challenging behaviour, which is not always valid. For example, a number of studies used the ABC-C total score, which is not valid.^66^ Fifth, many studies used an arbitrary cut-off score on behaviour rating scales to define challenging behaviour, and different studies used different scales and different cut-off scores. Thus, it is difficult to compare data among studies as it is difficult to know when data were pooled and whether all studies are describing the same challenging behaviour.

Deb & Hunter^61^ hypothesised that it is possible that underlying brain damage (in adults with severe and profound intellectual disabilities) and psychosocial factors (in those with mild intellectual disabilities) are stronger determinants of challenging behaviour than the presence of epilepsy. Factors that affect behaviour in the presence of epilepsy are related to: (a) underlying brain damage, such as the location and severity of any deformity, tumour or abnormal electrical discharge in the brain; (b) epilepsy-related factors, such as the presence of certain epileptic syndromes and genetic syndromes that are prone to lead to more challenging behaviour; (c) seizure-related factors, such as the severity, type and frequency of seizures; (d) anti-epileptic medication-related factors, such as the adverse effects of certain anti-epileptics and drug–drug interactions; and (e) psychosocial factors, such as loss of occupation, financial problems, lack of support, and locus of control being outside the person so the person does not have any control over the timing of seizures.

### Types of challenging behaviour

Many types of challenging behaviour were assessed in the included studies. The most common types that cause most concern and are most difficult to manage are aggression (verbal or physical aggression towards other people or property) and self-injurious behaviour. Pooled data showed a statistically significant higher rate of both aggression and self-injurious behaviour in the epilepsy group compared with the non-epilepsy group. It is worth remembering that, apart from epilepsy, many other medical and psychosocial factors influence these behaviours, including certain genetic syndromes that are known to be associated with aggression and self-injurious behaviour.^81^ Many of these syndromes also tend to predispose individuals to epilepsy (examples include Lesch–Nyhan and fragile-X syndromes).^1^ Therefore, it is difficult to draw any conclusion about the association between these specific challenging behaviours and epilepsy in isolation without considering all the other predisposing (e.g. genetic syndromes), precipitating (e.g. infection or anxiety) and perpetuating (e.g. inappropriate treatment and/or environment) factors for challenging behaviour in general. Pooled data did not show any significant intergroup difference in the rate of stereotyped behaviour. Only a small number of studies were involved in this meta-analysis and the heterogeneity level was high. Therefore, this finding has to be interpreted with caution.

The rates of other behaviours, such as inappropriate sexual behaviour, irritability, hyperactivity, verbal aggression, antisocial behaviours and lethargy, were reported in a very small number of studies showing equivocal findings. Therefore, it is difficult to draw any definitive conclusion about the association between these specific types of challenging behaviour and epilepsy. Another problem is that none of these specific behavioural types was defined using any standardised criteria (such as the Modified Overt Aggression Scale for rating aggression)^15^^,^^82^ but were based on single-item scoring.

### Association with epilepsy variables

When different subgroups according to various epilepsy variables were compared for the rate of challenging behaviour no clear picture emerged. In three studies^54^^,^^61^^,^^72^ the rate of challenging behaviour was significantly higher in those who had generalised seizures compared with those who had focal seizures, but in one study^67^ no significant difference was reported between these two groups. Given the small number of studies involving small numbers of participants in the subgroups, lack of matching of the groups, and different types of challenging behaviour rated in these studies, it is difficult to draw any definitive conclusion about any association between challenging behaviour and seizure type. Furthermore, Deb^62^ has shown that a high proportion of adults with intellectual disabilities who had a clinical diagnosis of primary generalised seizure showed focal epileptiform changes in their EEGs, thus raising the possibility that in many cases these generalised seizures are secondarily generalised from focal seizures.

Although three studies^38^^,^^61^^,^^72^ showed a significantly higher rate of challenging behaviour among those who had frequent seizures compared with those who had less frequent seizures, five studies^37^^,^^47^^,^^54^^,^^55^^,^^58^ did not find any significant intergroup difference. Therefore, it is difficult to draw any definitive conclusion about the influence of seizure frequency on the rate of challenging behaviour in this population. One confounder is the way frequency was rated in different studies, which varied widely. One study^61^ showed a significantly higher rate of challenging behaviour in those whose EEGs showed generalised epileptiform activities compared with those whose EEGs showed focal epileptiform changes. However, in another three studies^53^^,^^62^^,^^67^ no significant intergroup difference emerged between those with generalised as opposed to focal EEG changes. It is difficult to carry out an EEG for many adults with intellectual disabilities, particularly those who have severe and profound disability and also those who have challenging behaviour. EEG records are available for approximately 50–70% of adults with intellectual disabilities and 70–90% of these recordings are abnormal, although the abnormalities are not necessarily epileptiform in nature; rather, they present mostly as non-specific excess slow background activity.^62^

### Association with anti-epileptic medication

No definite association was found in the rate of challenging behaviour and polypharmacy with anti-epileptic medications. One would expect the participants in the polypharmacy group to have more severe epilepsy and, therefore, possibly more challenging behaviour. However, it is possible that anti-epileptic polypharmacy made these participants more sedated, thus dampening down the expression of challenging behaviour. In some people anti-epileptics improve both epilepsy symptoms and behaviour. However, in others it can have an opposite effect, in that although the epilepsy improves, the behaviour deteriorates.^9^ There may be many explanations for this paradoxical response. Old theories, such as forced normalisation^83^ or alternative psychosis,^84^ may provide some explanation. However, a more practical explanation may be that medication side-effects make the behaviour worse in some, despite improving epilepsy symptoms.

Although certain anti-epileptics, such as sodium valproate (restricted use in women of child-bearing age because of major worry about its teratogenicity), carbamazepine and lamotrigine, are known to improve mental state and are used to treat psychiatric disorders such as bipolar disorder,^85^ paradoxically some anti-epileptics are known to precipitate psychopathology, including challenging behaviour.^86^

Although the evidence is not strong, the available data suggest that the following anti-epileptic medications are likely to have some association with aggression and other challenging behaviours: phenobarbital, topiramate, vigabatrin, perampanel, zonisamide, levetiracetam, clobazam, clonazepam and tiagabine. Among these perhaps levetiracetam (aggression or agitation in 13% of treated patients), perampanel (in 12% at 8 mg/day and 20% at 12 mg/day) and possibly topiramate (in 2–10%) showed the strongest evidence for precipitating challenging behaviour.^86^ However, levetiracetam seems to improve behaviour in some adults with intellectual disabilities and worsen it in others.^87^ These anti-epileptic-related adverse effects may be more pronounced among adults with intellectual disabilities.^5^ However, it is clear that vast majority of those who receive these medications do not show any challenging behaviour. It is difficult to draw any definite conclusion from this review, as only one study reported the rate of challenging behaviour related to specific anti-epileptic drugs (59% of participants on carbamazepine, 55% on sodium valproate, 53% on phenytoin and 78% on lamotrigine monopharmacy showed challenging behaviour).

Although monopharmacy with anti-epileptic medication is desirable,^88^ polypharmacy with anti-epileptics is common in intellectual disability populations. Therefore, anti-epileptic drug–drug interaction are more likely, some of which may lead to challenging behaviour. Given that both antipsychotic and antidepressant medications are commonly prescribed among adults with intellectual disabilities,^89^ their interaction with anti-epileptics must be considered in any assessment of challenging behaviour. Also, both antipsychotics and antidepressants are likely to lower seizure threshold (particularly the older generation ones and at a high dose), which may precipitate more seizures and may lead to challenging behaviour.

Subgroup comparisons do not provide adequate power to detect clinically significant difference because of the small numbers involved in each subgroup and the lack of a control group. Also, in the subgroups there is no consistency in the types of challenging behaviour described, as some studies provided the rate of overall challenging behaviour, but others reported the rates of different types of challenging behaviour, such as aggression, self-injurious behaviour and stereotypy, making it difficult to amalgamate data from different studies. It will be necessary to conduct a much larger randomised controlled trial to recruit a reasonable number of participants in each subgroup to provide adequate power to detect clinically significant intergroup differences.

### Clinical significance of the findings

Having epilepsy can restrict social activities and wider social integration. A careful risk assessment is necessary to balance independence/quality of life and seizure-related risks (e.g. travelling alone, taking a bath, seizure-related injuries, unpredictability of the timing of seizures, SUDEP). The risk assessment should be part of the person's overall person-centred support plan and should be monitored and reviewed regularly with the person, their family/caregivers, other relevant professionals and the multidisciplinary team. One has to remember to mitigate against the impact on the family/caregivers. It is also important to remember that, apart from epilepsy, many circumstances, such as medical, psychological, social and environmental factors, affect the behaviour of someone with intellectual disabilities, and a full multidisciplinary person-centred assessment is required to develop an appropriate formulation for the management of challenging behaviour, including psychosocial interventions.^10^ Support staff, and the person and their family/carers, need to be informed of the risk factors (including SUDEP) and prognosis.^90^^–^^92^ This also highlights the requirement for regular health checks for all adults with intellectual disabilities, as highlighted in a recent NHS England publication.^93^

### Strengths

Our review received a high rating on the AMSTAR 2 quality control checklist for systematic reviews, as we complied with all of its requirements (supplementary Appendix 3). We carried out meta-analyses that were not done in any previous systematic reviews. We included a comprehensive Cochrane risk-of-bias table, which was not done by any of the previous systematic reviews. We included a much higher number of articles covering data from a much larger number of participants compared with the previous systematic reviews. We have registered our review with a well-established database, PROSPERO, so that our protocol is available for public scrutiny. This was not done by the previous reviews. We also carried out a very extensive hand-search of journals in the field of intellectual disability and epilepsy, along with stringent cross-referencing.

### Weaknesses

We searched for articles in English only. We excluded the grey literature and conference abstracts, as we felt it would be difficult to apply our eligibility criteria and risk-of-bias assessment on the basis of abstracts only. Although we used a stringent method for literature search, it is still possible that we missed some relevant articles. Our analysis showed conflicting evidence, in that the meta-analysis of pooled data from a larger number of studies did not show a significant intergroup difference, whereas pooled data from a smaller number of studies after sensitivity analysis showed a significant difference. Although we amalgamated data where possible to carry out a number of meta-analyses, the heterogeneity among studies remains high. It is difficult to amalgamate data from studies that used such diverse methodologies and defined challenging behaviour in so many different ways. Therefore, our findings must be interpreted with caution, as a lot of confounders could not be controlled for. However, to counteract the problem with study heterogeneity we used sensitivity analyses. Ideally, we should have used raw data for the meta-analysis, but this was not possible. Also, by log-transforming some data we may have lost some power in the meta-analysis.

### Research implications

Even though the meta-analysis of pooled data from a smaller number of studies after sensitivity analysis showed a significantly higher rate of challenging behaviour in the epilepsy group, the effect sizes are small, which may not be clinically significant. Also, there are major methodological flaws (highlighted by Cochrane risk-of-bias and SIGN 50 assessments) in the included studies. There is therefore a need for large-scale properly controlled studies. The included studies primarily concentrated on inter-ictal challenging behaviour. However, peri-ictally some people may show aggression, which is not goal directed but inadvertently may injure others. This might also be studied in further research.

## Data Availability

Data availability is not applicable to this article as no new data were created or analysed in this study.

## Supplementary material

For supplementary material accompanying this paper visit http://dx.doi.org/10.1192/bjo.2020.96. click here to view supplementary material
